# Parasites lacking the micronemal protein MIC2 are deficient in surface attachment and host cell egress, but remain virulent *in vivo*

**DOI:** 10.12688/wellcomeopenres.11594.2

**Published:** 2017-07-24

**Authors:** Simon Gras, Allison Jackson, Stuart Woods, Gurman Pall, Jamie Whitelaw, Jacqueline M. Leung, Gary E. Ward, Craig W. Roberts, Markus Meissner

**Affiliations:** 1 Wellcome Trust Centre For Molecular Parasitology, Institute of Infection, Immunity & Inflammation, Glasgow Biomedical Research Centre, University of Glasgow, Glasgow, G12 8TA, UK; 2 Strathclyde Institute of Pharmacy and Biomedical Sciences, Glasgow, G4 0RE, UK; 3 Department of Biology, Indiana University, Bloomington, IN, 47405, USA; 4 Department of Microbiology and Molecular Genetics, College of Medicine, University of Vermont, Burlington, VT, 05405, USA

**Keywords:** Toxoplasma, Microneme, Gliding motility, Host cell invasion, TRAP, MIC2, Plasmodium

## Abstract

*Background: *Micronemal proteins of the thrombospondin-related anonymous protein (TRAP) family are believed to play essential roles during gliding motility and host cell invasion by apicomplexan parasites, and currently represent major vaccine candidates against *Plasmodium falciparum*, the causative agent of malaria. However, recent evidence suggests that they play multiple and different roles than previously assumed. Here, we analyse a null mutant for MIC2, the TRAP homolog in *Toxoplasma gondii*. *Methods: *We performed a careful analysis of parasite motility in a 3D-environment, attachment under shear stress conditions, host cell invasion and *in vivo *virulence. *Results: *We verified the role of MIC2 in efficient surface attachment, but were unable to identify any direct function of MIC2 in sustaining gliding motility or host cell invasion once initiated. Furthermore, we find that deletion of *mic2* causes a slightly delayed infection *in vivo,* leading only to mild attenuation of virulence; like with wildtype parasites, inoculation with even low numbers of *mic2 *KO parasites causes lethal disease in mice. However, deletion of *mic2 *causes delayed host cell egress *in vitro*, possibly via disrupted signal transduction pathways. *Conclusions: *We confirm a critical role of MIC2 in parasite attachment to the surface, leading to reduced parasite motility and host cell invasion. However, MIC2 appears to not be critical for gliding motility or host cell invasion, since parasite speed during these processes is unaffected. Furthermore, deletion of MIC2 leads only to slight attenuation of the parasite.

## Introduction

Apicomplexan parasites are obligate intracellular parasites that invade the host cell in an active process that involves the parasite’s own acto-myosin system acting in concert with parasite-derived surface ligands ( Meissner *et al.*, 2013). These ligands are derived from secretory organelles, the micronemes, which are unique to apicomplexan parasites. Indeed, microneme secretion has been demonstrated in several studies to be linked to efficient parasite invasion and gliding motility, and it has been suggested that micronemal proteins act as force transmitters for the acto-myosin system, similar to the role of integrins in amoeboid cells ( Bargieri *et al.*, 2014; Tardieux & Baum, 2016). These proteins are then cleaved by rhomboid proteases (ROMs) to release the force ( Rugarabamu *et al.*, 2015; Shen *et al.*, 2014a).

One crucial family of micronemal proteins is the thrombospondin-related proteins, such as TRAP, MTRAP, TSP and CTRP, which have been suggested to be essential for gliding motility and invasion in diverse life stages of *Plasmodium* spp. ( Bartholdson *et al*., 2012; Baum *et al.*, 2006; Dessens *et al*., 1999; Morahan *et al.*, 2009; Moreira *et al*., 2008; Sultan *et al*., 1997). Similarly, the *Toxoplasma gondii* homolog of TRAP, MIC2, is thought to be required for gliding motility and invasion, and a conditional knockdown mutant for *mic2* suggested that MIC2 is an important component of this machinery ( Huynh & Carruthers, 2006). However, recent results questioned the importance of MIC2, since it is relatively straightforward to obtain clonal *mic2* null mutants using reverse genetic tools, such as a conditional recombinase system ( Andenmatten *et al.*, 2013). Furthermore, a recent genome-wide CRISPR/Cas9 screen indicated a relatively minor contribution of *mic2* to parasite fitness *in vitro* ( Sidik *et al.*, 2016). Given the huge repertoire of micronemal proteins, it is thus tempting to speculate that multiple redundancies exist among these proteins. Such a situation has been described for AMA1, a micronemal protein that is involved in host cell invasion, but is not essential ( Bargieri *et al.*, 2013; Lamarque *et al.*, 2014).

A recent systematic dissection of other proteins involved in gliding motility, such as parasite actin, myosin A and its light chain MLC1, GAP45 and GAP40 has identified novel functions for these proteins and demonstrated that the gliding machinery is involved in the formation and release of attachment sites ( Bargieri *et al.*, 2014; Egarter *et al.*, 2014a; Harding *et al.*, 2016; Periz *et al.*, 2016; Whitelaw *et al.*, 2017). Furthermore, MIC2 is thought to interact with parasite F-actin via a connector protein that has been recently described and suggested to bind both the cytosolic tail of MIC2 and parasite F-actin ( Jacot *et al.*, 2016), which might well be involved in the regulation of attachment sites.

Given our evolving understanding of parasite motility mechanisms, we set out to re-analyse the functions of *mic2* during the parasite’s asexual life cycle, both *in vitro* and *in vivo*. We confirm previous findings, demonstrating that MIC2 is involved in gliding motility and invasion ( Huynh & Carruthers, 2006). This involvement is best demonstrated in attachment assays that suggest an important role for MIC2 in the generation of attachment sites that are required for efficient motility. However, while *mic2*KO parasites exhibited less motility and move on shorter distance, they reach the same maximal speeds as WT parasites. Similarly, fewer parasites lacking MIC2 invade host cells, but when they do invade they do so at speeds comparable to WT parasites. Like parasites deficient in other components of the acto-myosin system, with which MIC2 interacts, the *mic2* KO parasites show delayed host cell egress. Mice have been widely use and an *in vivo* model of toxoplasmosis to test virulence of *T. gondii* in mammalian hosts. Unexpectedly, *in vivo* analysis demonstrates that they are only mildly attenuated and still induce lethal disease in mice. RNA sequencing analysis revealed that deletion of *mic2* has a minor impact on the transcription levels of other micronemal, ROMs and motor complex proteins, as well as several proteins with no obvious connection to invasion or motility, suggesting a multifactorial adaptation to loss of MIC2.

## Results

### mic2 is an important but non-essential gene

**Figure 1.**
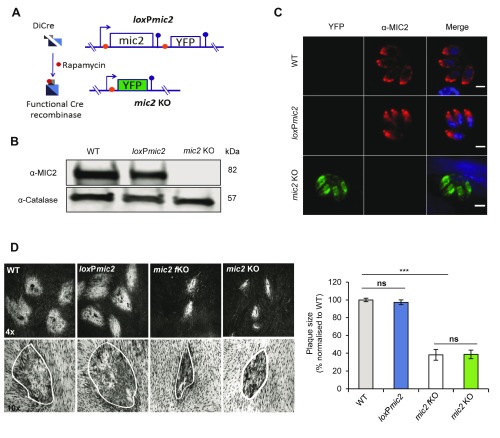
Generation of a *mic2*KO clonal line using DiCre recombinase system. **A**) Schematic representation of the construct used for generating *mic2* KO parasites. The endogenous *mic2* was replaced by *mic2* cDNA flanked with loxP sites (orange circles). Upon addition of rapamycin, the gene was excised and YFP expressed. Stop codons are represented by blue circles. **B**) Immunoblot analysis of MIC2 expression in WT, *lox*P *mic2* and *mic2* KO parasites. Catalase was used as a loading control. **C**) IFA of MIC2 and YFP expression in WT, *lox*P *mic2* and *mic2* KO parasites. Scale bars: 2 µm. n=6, total number of vacuoles observed >450. **D**) Representative examples and analysis of plaque assays comparing WT, *lox*P *mic2, mic2* FKO and *mic2* KO. *** *p*-value <0.001 in a two-tailed Student's t-test.

We previously described the generation and initial characterisation of a null mutant for *mic2* using the DiCre system ( Andenmatten *et al.*, 2013). In this mutant, the native *mic2* gene has been replaced by loxP-flanked *mic2* cDNA ( *lox*P *mic2*). Upon Cre-mediated site-specific recombination, the *mic2* cDNA was removed, and the reporter gene *YFP* placed under the control of the endogenous promoter, resulting in green fluorescent *mic2* KO parasites ( Figures 1 A–C). Despite forming smaller plaques when compared to the WT RH strain, *mic2* KO parasites can be easily isolated and maintained in culture, demonstrating that *mic2* is an important, but non-essential gene in the *Toxoplasma* lytic cycle, as described previously ( Andenmatten *et al.*, 2013). No significant differences in plaque size were observed between *lox*P *mic2* and WT *T. gondii* parasites ( Figure 1D). To address the possibility of adaptation to *mic2* loss, we compared freshly induced *mic2* KO (1 lytic cycle, *mic2* FKO) to *mic2* KO parasites cultured for more than one year after induction. No significant difference in plaque size was observed between the two strains ( Figure 1D). Proteolytic processing and trafficking of the MIC2-associated protein (M2AP) was previously shown to depend on MIC2 ( Huynh & Carruthers, 2006). We confirmed that depletion of MIC2 leads to M2AP mislocalisation and constitutive secretion of unprocessed M2AP ( Figure S1) with no significant effects on the localisation or secretion of other tested micronemal proteins.

### Deletion of mic2 leads to only minor changes in the expression of other known invasion machinery components

**Figure 2.**
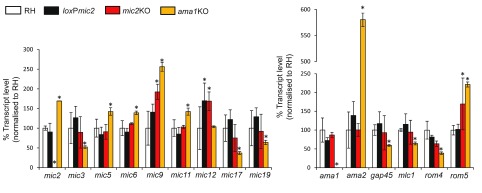
Transcriptional analysis of MIC expression levels in the *mic2* KO parasites. Graphical representation of percentage of mean FPKM value normalised to RH value for RH (White), *lox*P *mic2* (Black), *mic2* KO (Red) and *ama1*KO (Yellow) strains. Differences between each mutant and RH were calculated using CutDiff with a comparison of three independent biological replicates, using the quartile library normalization method, a “pooled” dispersion estimation method with the three replicates and a false discovery rate of 0.05. Statistically significant differences from RH are indicated by *. Error bars indicate the FPKM standard deviation within the replicate.

In the case of *ama1,* the removal of the gene leads to the upregulation of its homologue *ama2*, allowing compensation of the loss of *ama1* function ( Bargieri *et al.*, 2013; Lamarque *et al*., 2014). To test if removal of *mic2* leads to up- or downregulation of known components of the invasion machinery (i.e. other micronemal or glideosome proteins, actin, etc.), RNA sequencing analysis was performed to compare relative gene expression levels in RH, *lox*P *mic2*, *mic2* KO and *ama1* KO. RH was used as a reference (100%) ( Figure 2, Figure S2). In parallel, we confirmed for *ama1* KO that transcription levels of *ama2* were upregulated. We also observed increased transcription levels of various other genes in *ama1* KO that are implicated in invasion, such as *mic2*, *mic5, mic6*, *mic9*, *mic11*, and *rom5,* as well as downregulation of *mic3*, *mic17a*, *mic19*, *gap45, mlc1* and *rom4,* suggesting that adaptation to *ama1* disruption in culture is multifactorial. Although *ama2* shows the strongest upregulation, the overall expression level of *ama2* remains very low compared to *ama1* ( Figure S2, Table S1).

Fewer differences were seen between *lox*P *mic2* and *mic2* KO parasites than were seen in *ama1* KO ( Figure S2, Table S1). Other than *mic9* and *rom5,* deletion of *mic2* had little effect on the transcription level of the known MICs, glideosome or rhomboid genes examined. Furthermore, when we compared the whole transcriptomes, we observed similar changes in *ama1* KO and *mic2* KO, suggesting a potential multifactorial and overlapping adaptation process. The genes whose expression changed include several proteases, surface antigens and hypothetical proteins ( Table S2 and Table S3). However, based on this analysis we were unable to identify a clear candidate that could compensate for los of *mic2*.

### Attachment is impaired in mic2 KO parasites

**Figure 3.**
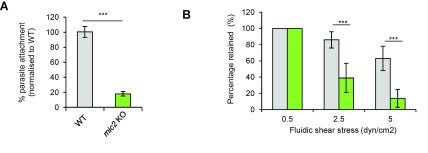
*mic2*KO parasites show impaired attachment to host cells and collagen IV. **A**) Percentage of parasites attached to host cells after 30 min. Mean values of three independent assays are shown ± SEM, *** *p*-value <0.001 in a two-tailed Student's t-test. **B**) Percentage of parasites retained on a collagen IV-coated surface under fluidic shear stress relative to 0.5 dyn/cm ^2^ flow for RH and *mic2* KO. Mean values of three independent assays are shown ± SEM, *** *p*-value <0.001 in a two-tailed Student's t-test.

We previously demonstrated that the acto-myosin system of the parasite is important for surface attachment and required for efficient initiation of gliding motility ( Whitelaw *et al.*, 2017). Since MIC2 is connected to this system, we wished to investigate if a similar phenotype can be observed in *mic2* KO parasites. Using a standard attachment assay ( Whitelaw *et al*., 2017), we observed that the percentage of *mic2* KO parasites attached to host cells dropped to 20% in comparison to WT ( Figure 3A), consistent with previous studies ( Harker *et al*., 2014; Huynh & Carruthers, 2006). We also estimated attachment strength using a fluidic shear stress assay on surfaces coated with collagen IV ( Figure 3B). Parasites were incubated without flow to allow them to attach to the surface. An initial flow of 0.5 dyn/cm ^2^ was applied to wash off unattached parasites and the remaining number of parasites for each strain was considered as the initial value (100%). In contrast to control parasites, *mic2* KO parasites were washed off rapidly at low shear stress (39% *vs.* 86.6% parasites remaining for *mic2* KO and WT, respectively at 2.5 dyn/cm ^2^), consistent with previous results using the *mic2* conditional knockdown ( Harker *et al*., 2014). In summary, *mic2* KO parasites have a deficiency in their capacity for surface attachment, which will inevitably affect gliding motility and host cell invasion.

### Depletion of MIC2 has little impact on gliding speed

**Figure 4.**
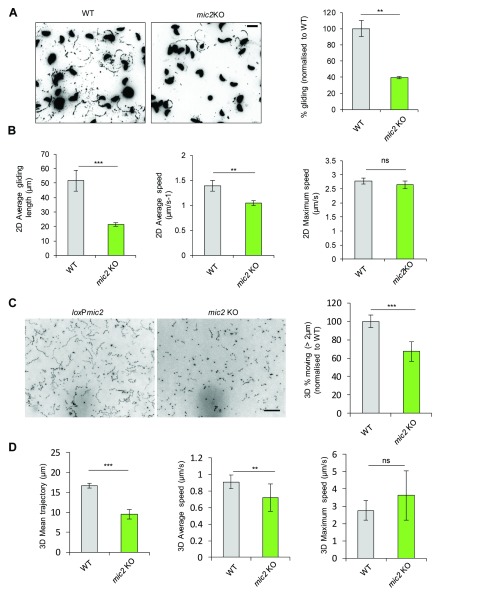
Gliding initiation and gliding distance are affected by *mic2* KO attachment defect but not maximal speed. **A**) Trail deposition assay of WT parasites compared to *mic2* KO. Mean values of three independent assays are shown ± SEM and ** *p*-value <0.01 in a two-tailed Student’s t-test. Scale bar 5 μm **B**) Kinetic analysis of 2D helical gliding. Data were analysed using auto-tracking software. Mean values of three independent assays are shown ± SEM, ***: *p*-value <0.001 in a two-tailed Student’s t-test. **C**) Representative maximum intensity projections of 3D Matrigel-based motility assays comparing WT to *mic2* KO parasites (left) and % of parasites moving (right) normalised to WT. Scale bar 50 μm **D**) Analysis of 3D trajectories; mean values of three independent assays are shown ± SD, ***: *p*-value <0.001 in two-way Anova with Sidak’s multiple comparisons test.

To analyse the role of MIC2 during gliding motility, we performed standard trail assays to determine the ratio of gliding to immotile parasites. Using this analysis, we confirmed that the majority (~60%) of *mic2* KO parasites are incapable of initiating gliding compared to WT ( Figure 4A), as previously reported ( Huynh & Carruthers, 2006). By video microscopy, the three types of motility (circular, helical and twirling) ( Håkansson *et al.*, 1999) were observed ( Video S1– Video S6). Time lapse analysis showed that once motility was initiated, depletion of MIC2 has an effect on both the average speed and average distance travelled by helically gliding parasites, while the maximal speed was not affected ( Figure 4B). In the case of circularly gliding parasites, a reduction in the average distance travelled was seen for *mic2* KO parasites, consistent with previous data ( Huynh & Carruthers, 2006). Surprisingly, the average and maximum speeds of circular gliding increased compared to WT parasites ( Figure S3).

Next, we wished to investigate gliding motility in a more physiological 3D environment ( Figure 4C). In good agreement with the 2D-motility assays, fewer *mic2* KO parasites initiated motility in a 3D matrix when compared to WT parasites. Furthermore, a significant reduction in average displacement (9.54 ± 1.17 µm *vs.* 16.66 ± 0.58 µm) and average speed (0.72 ± 0.16µm/s ^-1^ *vs.* 0.91 ± 0.08 µm/s ^-1^) was observed in *mic2* KO *vs.* WT parasites, respectively ( Figure 4D). Again, no reduction in maximal speed was observed, indicating an “all-or-nothing” response ( Figure 4D). Together, these data suggest that, similar to MyoA, MLC1 and F-Actin, the predominant function of MIC2 is in the establishment of attachment sites required for effective initiation of motility.

### Depletion of MIC2 results in lower invasion rates, but has no influence on invasion speed

**Figure 5.**
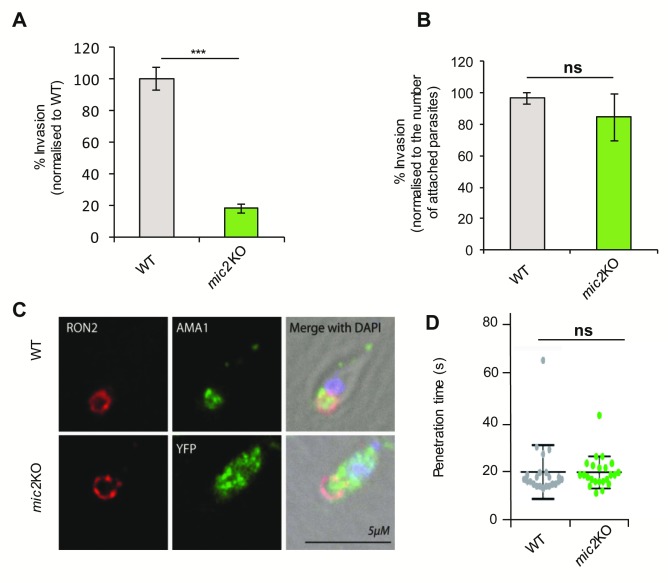
The *mic2* KO defect in invasion is due to diminished attachment capacity. **A**) *mic2* KO and WT parasites were incubated for 1h with HFF cells, and invasion rate was calculated by comparing the number of *mic2* KO *vs.* WT parasites invaded. Mean values of three independent assays are shown ± SEM, ***: *p*-value <0.001 in a two-tailed Student's t-test. **B**) Normalised invasion assays. For each strain ( *mic2* KO and *WT*), the number of invaded parasites was normalized to the total number of parasites observed (attached + invaded). Mean values of three independent assays are shown ± SEM. **C**) IFA of the junction protein RON2 and AMA1/YFP in *mic2* KO and WT tachyzoites, scale bar 5 μm. n=3, total number of parasites observed >50. **D**) Penetration kinetics of *mic2* KO and WT tachyzoites determined by time-lapse microscopy (n=25).

Next, we investigated the invasion process of *mic2* KO parasites ( Figure 5A) and found, as previously described ( Huynh & Carruthers, 2006), that invasion is strongly inhibited, at 18 ± 3% relative to WT invasion levels. In this assay the overall failure of invasion is measured, which could be due to defects in host cell attachment, junction formation or host cell penetration. To differentiate between these individual steps, invasion rates were normalized to the total number of interacting parasites ( *i.e.*, attached plus invaded). Interestingly, in this case the invasion rates of WT and *mic2* KO parasites are similar, demonstrating that the reduced invasion of *mic2* KO parasites relative to WT parasites is mainly due to their failure to attach to the host cell ( Figure 5B). Next, we assessed junction formation and penetration speeds ( Figures 5C and D) and were unable to detect significant differences between WT and *mic2* KO parasites. *mic2* KO parasites invaded through a normal junction and penetrated the host cell at speeds similar to those of WT parasites (21.3 ± 11.7 s and 21.0 ± 6.9 s for WT and *mic2* KO, respectively) ( Video S7 and Video S8). These results lead us to conclude that the invasion deficiency observed for *mic2* KO parasites is due to impaired attachment to the host cell, as suggested previously ( Huynh & Carruthers, 2006).

### Effect of mic2 deletion on intracellular parasites

**Figure 6.**
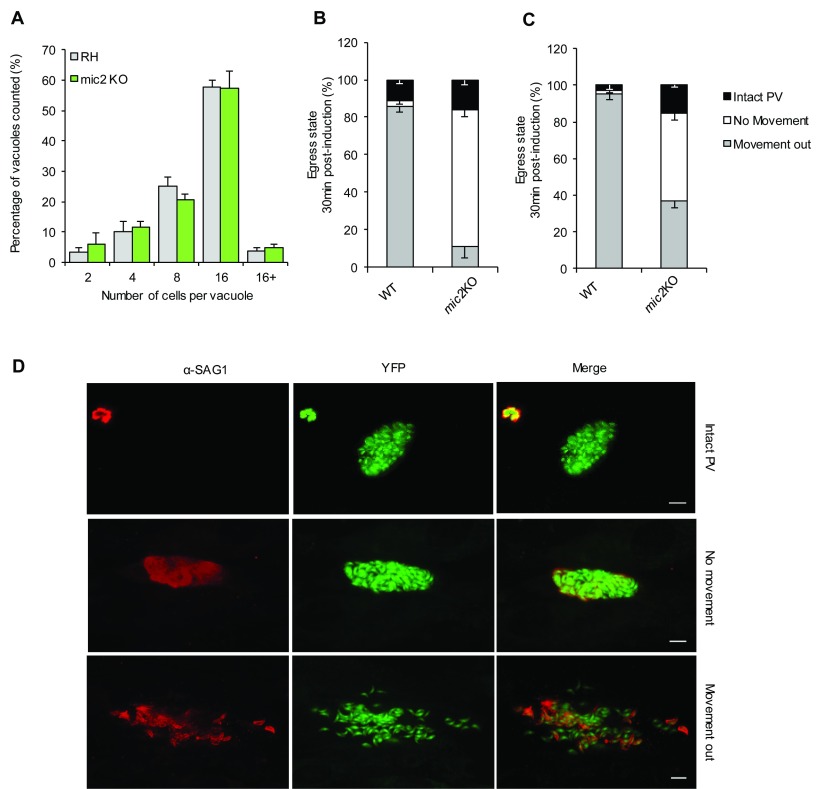
Parasites lacking MIC2 replicate normally, but are defective in host cell egress. **A**) Replication analysis of *mic2* KO parasites. Parasites were allowed to invade for 1 h prior to intracellular growth for 24 h and the number of parasites per parasitophorous vacuole was counted. Mean values of three independent assays are shown ± SEM. **B** and **C**) Parasite egress was artificially induced with Ca2+ ionophore (A23187) for 10 ( **B**) and 30 min ( **C**). For quantification, three outcomes were scored: parasites failed to lyse the vacuole (Intact PV), parasites lysed the vacuole but did not move (No movement) or classical egress (Movement out). Mean values of three independent egress assays are shown ± SEM. **D**) IFA illustrating a newly lysed vacuole (30 minutes post-induction) where antibody against SAG1 can only access part of the vacuole (top panels) and two fully lysed vacuoles, one showing little to no movement of *mic2* KO parasites out of the vacuole (middle) and the other showing normal egress (bottom) Scale bar 5 μm.

We also readdressed the function of MIC2 in intracellular development and egress. While intracellular replication of *mic2* KO parasites appeared normal ( Figure 6A), disruption of *mic2* caused a significant delay in host cell egress ( Figure 6B–D). When egress was artificially triggered with a Ca ^2+ ^ionophore (A23187), *mic2* KO parasites were able to rupture the parasitophorous vacuole at levels comparable to WT (89 ± 2 % *vs.* 84 ± 5% for WT and *mic2* KO respectively) ( Figure 6B). However, a higher proportion of *mic2* KO ** parasites were unable to leave the host cell after lysing the parasitophorous vacuole membrane, suggesting a defect in initiating motility ( Figure 6C and D, and Video S9– Video S10). This defect was still evident even at 30 min after induction, as only 37 ± 3 % of *mic2* KO had moved out of the vacuole compared to 95 ± 3 % for WT.

### mic2 KO parasites are less virulent than WT parasites but still lethal in mice

**Figure 7.**
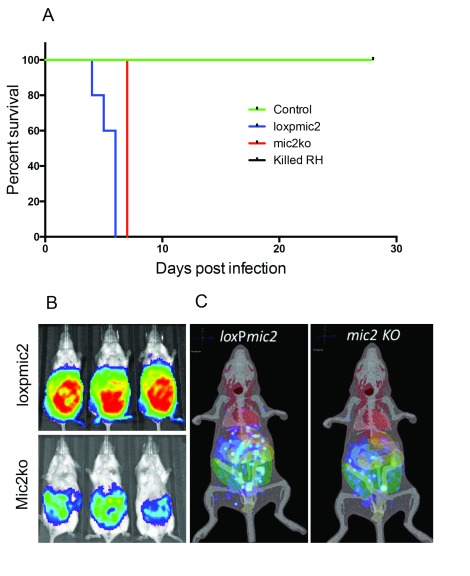
mic2 KO parasites induce lethal disease in mice and have a similar distribution in infected mice as WT parasites. **A**) Mice were infected with 2×10 ^4^ tachyzoites intraperitoneally with PBS, killed *T. gondii* RH strain or mic2KO RH strain *T. gondii* and disease followed. Mice infected with the RH strain of *T. gondii* succumbed to infection by day 7 post infection. **B**) The localisation of *lox*P *mic2 T. gondii* RH strain parasites and *mic2* KO *T. gondii* strain parasites transfected with luciferase were broadly similar at day 5 post infection with parasites evident predominantly in the peritoneal cavity. Heat map represents the intensity of the detected luciferase signal. **C**) This localisation was supported by 3D diffuse tomographic reconstruction.

Previous data indicated that *mic2* knockdown parasites are avirulent in mice ( Huynh & Carruthers, 2006). To test whether this is also the case for the *mic2* KO ** parasites, mice were infected intraperitoneally with *mic2* KO, *lox*P *mic2,* killed WT tachyzoites (1×10 ^4^) or PBS. The *mic2* KO parasites caused severe disease in mice leading to death or necessitating their euthanasia at humane endpoints by day 7 ( Figure 7A). To verify that the cause of severe disease was due to parasite replication and to allow visualization of parasite burden in real time *in vivo*, we generated *mic2* KO parasites stably expressing a red shift luciferase. Five days post-infection, the *mic2* KO parasites were observed at similar anatomical locations, but with a less heavy parasite burden compared to similarly transfected *lox*P *mic2* parasites ( Figures 7B and C).

## Discussion

Gliding motility and host cell invasion by apicomplexan parasites have been thought to critically depend on members of the thrombospondin-related anonymous protein (TRAP) family, which are transmembrane proteins derived from the micronemes ( Huynh & Carruthers, 2006; Sultan *et al.*, 1997). A huge body of research on these proteins led to the widely accepted model that they act as a link between the parasite’s cytoskeleton and the host cell by binding to surface receptors with their extracellular domain, and to parasite aldolase, which in turn interacts with parasite actin, via their C-terminal domain ( Jewett & Sibley, 2003; Morahan *et al.*, 2009). Aldolase was recently shown to be dispensable for gliding motility and invasion ( Shen & Sibley, 2014b), but a new “connector” protein postulated to link the tail of MIC2 to actin has been described ( Jacot *et al*., 2016). Nevertheless, recent re-analysis of TRAP mutants suggested that these proteins do not necessarily function during motility as force transmitters, but rather in the regulated formation and release of adhesion sites ( Hegge *et al.*, 2010; Münter *et al.*, 2009), since *Plasmodium* sporozoites remain motile in the absence of TRAP or TLP and can be chemically complemented on tuneable substrates ( Hellmann *et al.*, 2013). Furthermore, other members of the TRAP family have been demonstrated to have unexpected functions, unrelated to gliding motility or invasion. For example MTRAP has long been seen as the merozoite specific TRAP homolog that is required for merozoite invasion ( Baum *et al.*, 2006), but recent studies demonstrate that its crucial function lies in gametocyte egress ( Bargieri *et al.*, 2016; Kehrer *et al.*, 2016). Furthermore, reassessment of other components of the gliding machinery in *T. gondii*, such as actin or MyoA, demonstrated that they play a crucial role in the formation of attachment sites, but not necessarily in force production *per se* ( Whitelaw *et al*., 2017), and our current view of the mechanics of this complex system requires further analysis ( Tardieux & Baum, 2016).

In the case of the TRAP-family protein MIC2, previous attempts to knock out the gene failed, suggesting an essential function ( Huynh & Carruthers, 2006). A conditional knockdown mutant was therefore generated and used to demonstrate important roles for MIC2 in gliding motility, attachment to host cells, host cell invasion and virulence *in vivo* ( Huynh & Carruthers, 2006). Using a conditional recombination system, however, we showed that it was possible to generate clonal null mutants for *mic2* ( Andenmatten *et al.*, 2013), demonstrating that MIC2 is not an essential gene. This finding was corroborated by a recent genome-wide phenotypic screen based on CRISPR/Cas9, indicating that disruption of *mic2* has only a mild phenotypic defect (phenotypic score = -1.17; ( Sidik *et al.*, 2016)).

Here, we assessed the functional consequences of deleting *mic2.* In contrast to other conditional null mutants generated using the DiCre system, such as the *myoA* KO, the isolation of clonal *mic2* KO mutants was straightforward, indicating only minor competition between non-induced ( *mic2 ^+^*) parasites and *mic2* KO parasites (data not shown). Interestingly, we could not identify any long-term phenotypic adaptation due to prolonged culturing of *mic2* KO parasites, since the phenotypes appear to remain unchanged over time. When we performed a comparative transcriptomic analysis, we found that deletion of *mic2* had little effect on the transcription level of known MICs, motor or ROM proteins, while multiple proteases (lipases, methionine aminopeptidase), an uncharacterized EGF and PAN domain containing protein, SAG-related proteins and hypothetical proteins were found to be slightly upregulated in the *mic2* KO ( Table S2). These data suggest that the deletion of *mic2* may result in a multifactorial adaptation that involves small differences in expression levels of seemingly unrelated genes. How rapidly such an adaptation occurs is not known; it is possible that the presence of other microneme proteins with partially overlapping functions immediately enables the parasite to tolerate the loss of *mic2*. Intriguingly, many of the seemingly unrelated genes upregulated in *mic2* KO were also upregulated in *ama1* KO ( Table S2 and Table S3).

Like the parasites depleted of MIC2 by conditional knockdown ( Huynh & Carruthers, 2006), *mic2* KO parasites showed no defect in the trafficking and localization of other microneme proteins (with the notable exception of M2AP) and no effect on intracellular replication, but dramatic effects on host cell invasion, which are due primarily to decreased attachment. Both the conditional knockdown and the KO showed that the loss of MIC2 results in reduced 2D motility, likely through an effect on attachment, and that helical motility is more affected than circular motility. We have extended the motility analysis and shown that in both 2D and 3D, the defect appears to be one of motility initiation; once *mic2* KO parasites start moving, they can reach the same maximal speeds as WT parasites. Interestingly a recent study compared adhesion of *mic2* knockdown parasites under fluidic stress and concluded that only initial attachment, but not strengthening of attachment sites was affected ( Harker *et al*., 2014). The invasion phenotype of the *mic2* KO is similar to that of *ama1* KO parasites, which also show reduced attachment to host cells, but penetration into the host cell at speeds similar to that of WT parasites ( Bargieri *et al.*, 2013). It remains to be seen if redundant proteins can compensate for gliding/invasion motility, but NOT attachment in the absence of MIC2, as suggested for AMA1 ( Lamarque *et al.*, 2014).

In contrast to the MIC2 conditional knockdowns ( Huynh & Carruthers, 2006), we find that *mic2* KO parasites have a significant delay in host cell egress. The reduced egress is not likely due to a complete inability of the parasite to move, since kinetic analysis of *mic2* KO parasites demonstrates only partial motility defects in a 2D and 3D environment. Rather it appears that motility is initiated with a significant delay, and that the parasites stay connected to each other (data not visualised). Future experiments will be required to elucidate the role of MIC2 during egress in more detail and to determine whether the reduced ability of *mic2* KO parasites to egress compared to the conditional knockdowns is due to residual expression of MIC2 in the conditional knockdown or to some other effect.

Most surprisingly, and in contrast to the results obtained for the MIC2 conditional knockdown ( Huynh & Carruthers, 2006), we find that *mic2* KO parasites are only mildly attenuated compared to WT RH parasites. The *mic2* KO line (at least at the doses used in these studies) induces lethal disease in BALB/c mice. One explanation for the different findings could be that RH TATi ΔHX, the parasite line used to generate a knockdown for *mic2* is already severely attenuated due to the expression of the Tet-transactivator. Indeed, in the study by Huynh & Carruthers, 2006, high doses (5 × 10 ^4^ tachyzoites) with RH TATi ΔHX were required to achieve normal time-to-death kinetics. It is thus possible that knockdown of *mic2* in this strain reflects an enhancement of the already attenuated phenotype, while depletion in a WT background has only mild effects on parasite virulence. Alternatively, in our studies a reduced virulence of mic2KO parasites is not evident because of the relatively high numbers of tachyzoites used to facilitate *in vivo* imaging. Consequently to fully compare the virulence LD50 studies would be required. Nonetheless our studies demonstrate unequivocally that MIC2 is not necessary for *in vivo* infection and lethality in BALB/c mice. *In vivo* imaging and 3D diffuse tomographic reconstruction of mice infected with MIC2 deficient parasites transfected with a luciferase gene demonstrated that they grow in similar anatomical locations in mice as control parasites. However, dissection and histopathological analyses would be necessary to rule out any potential differences in tissue trophism.

In summary, we find here that MIC2 acts as important, but not essential, attachment factor, and that reduced invasion and gliding rates are due to a decreased ability to initiate rather than to sustain motility. This is similar to the findings for other components of the acto-myosin system, such as actin, MyoA or MLC1, where it appears that formation of adhesion sites is one of the critical functions of this complex machinery ( Whitelaw *et al*., 2017). Finally, our finding that deletion of *mic2* causes only a mild attenuation of virulence *in vivo* will have implications for future vaccine design.

## Materials and methods

**Cloning DNA constructs:** All primers used in this study were synthesised by Eurofins (UK) and are listed in Table S4. A red shift luciferase (Bruce Branchini, Connecticut College, USA) was amplified using Luc fw/rv primers and cloned under the p5RT70 promoter with a chloramphenicol resistant cassette.

**Mic2 inducible KO vector:** As previously described ( Andenmatten *et al.*, 2013), to generate *loxPMic2loxP-YFP*- *HX*, the *mic2* 3′ UTR was amplified from genomic DNA using the primer pair 3′ UTR Mic2 fw/rv, and the PCR fragment was cloned into p5RT70loxPKillerRedloxPYFP-HX via SacI. The *mic2* ORF (TGME49_201780) was amplified from cDNA using the primers Mic2 ORF fw/rv and was cloned into the parental vector p5RT70loxPKillerRedloxPYFP-HX using EcoRI and PacI. Finally, the *mic2* 5′ UTR containing the endogenous promoter was amplified from genomic DNA using the primer pair 5′ UTR Mic2 fw/rv and cloned into the final vector using ApaI and EcoRI.

**Culturing of parasites and host cells:** Human foreskin fibroblasts (HFFs) were grown on TC treated plastics plates and maintained in Dulbecco’s modified Eagle’s medium (DMEM) supplemented with 10% foetal bovine serum (FBS), 2 mM L-glutamine and 25 mg/ml gentamycin. Parasites were cultured in HFFs and maintained at 37°C and 5% CO _2_.

***T. gondii* transfection and selection:** The conditional *mic2* knockout strain ( *ku80::diCre/endogenous mic2::loxPmic2loxP*, referred to here as loxPMic2) was generated as previously described ( Donald *et al.*, 1996) by transfecting 60 μg of the plasmid *loxPMic2loxPYFP-HX* into the *ku80di::Cre* parasites to replace the endogenous copy of *mic2*, and parasites containing stable integration of this construct were selected using xanthine and mycophenolic acid, as previously described ( Donald *et al.*, 1996). The resulting loxPMic2 strain carries only one copy of *mic2*, which can be excised by adding rapamycin (50 nM in DMSO for 4 h before washout) to generate the *mic2* null mutant ( *ku80::diCre/mic2* ^−^, referred to here as *mic2* KO). The clonal *mic2* KO line was isolated by performing serial dilutions on the clonal induced loxPMic2 strain after 4 h induction and subsequent removal of rapamycin. After verification of the integration, protein expression was checked by western blotting using anti-MIC2 and anti-catalase antibodies and IFA using anti-MIC2 antibodies and YFP expression. Red shift luciferase expressing *lox*P *mic2* and *mic2* KO were obtained by transfecting a Red-shift luciferase expressing plasmid using random integration. Parasites were then selected for chloramphenicol resistance and luciferase expression.

**RNA extraction and sequencing:** RH, *lox*P *mic2*, *mic2* KO and *ama1* KO RNA was extracted using the RNeasy ^®^ Mini Kit (Qiagen) in a biological triplicate. Eluate RNA concentration was determined by Nanodrop (Thermo Scientific). For sequencing, 4ug per sample was sent to NGS Laboratory, Glasgow Polyomics (University of Glasgow, Bearsden, UK). RNA was analysed after polyA library preparation using paired-end with a depth of sequencing of 25M bases on a Next Seq 500 sequencer. Results were analysed on Galaxy server, with software provided ( http://heighliner.cvr.gla.ac.uk/login?redirect=%2F). After trimming, data were aligned to the *T. gondii* genome using TopHat2. After mapping, differential expression compared to RH was determined using Cutdiff. For each analysis, data for the three biological triplicates were carried out under the same condition ( *lox*P *mic2*, *mic2* KO, *ama1* KO) and compared to the triplicate of RH parasites using a quartile library normalization method, a pooled dispersion estimation method with a false discovery rate of 0.05. Individual FPKM of each sample was also extracted to control the analysis. 

**Immunofluorescence analysis:** IFA was carried out as previously described ( Egarter *et al.*, 2014b). Briefly, parasites were allowed to invade and replicate in a HFF monolayer grown on glass coverslips. The intracellular parasites were fixed in 4% paraformaldehyde for 20 min at room temperature (RT). Afterwards coverslips were blocked and permeabilised in 2% BSA and 0.2% Triton X–100 in PBS for 20 min at RT. The staining was performed using the indicated combinations of primary antibodies for 1 h at RT, followed by the incubation with AlexaFluor 350-, AlexaFluor 488-, AlexaFluor 594- or AlexaFluor 633-conjugated secondary antibodies (1:3000, Invitrogen–Molecular Probes) for another 45 min at RT. For a list of all antibodies used in this study see Table S5.

**Western-blot:** Freshly egress RH, *lox*P *mic2* and *mic2* KO parasites were harvested, filtered and washed before being resuspendend in PBS containing Pierce™ Protease Inhibitor Mini Tablets, EDTA Free (Thermo Scientific) and Triton X-100 0.2%. Western blots were processed using the indicated combination of primary antibodies for 1h at RT, followed by three washes and incubation with IRDye LiCor secondary antibodies (680RD and 800W, 1:20 000) for another hour at RT. Labeled membranes were visualized using Li Cor Odyssey Clx. For a list of all antibodies used in this study see Table S5.

### Phenotypic characterisations

**Plaque assay:** Plaque assays were conducted as described previously ( Egarter *et al.*, 2014b). 1×10 ^3^ parasites were inoculated on a confluent monolayer of HFFs and incubated for 5 days at 37°C and 5% CO _2_, after which the HFFs were washed once with PBS and fixed with ice cold MeOH for 20 minutes. HFFs were stained with Giemsa with the plaque area measured using Fiji software version 1.8.0_66 ( https://fiji.sc/). Mean values of three independent experiments +/- SD were determined.

**Secretion assays:** Microneme secretion was assayed by monitoring the release of MIC2, M2AP and MIC4 into the culture medium, as described previously ( Huynh & Carruthers, 2006). Secretion was observed in absence (constitutive secretion) or presence of 2 μM A23187 (induced secretion) at 37°C.

**Attachment assay:** 1 ×10 ^6^ parasites were allowed to invade a confluent monolayer of HFF cells for 10 min. Cells were washed and fixed with cold 4% PFA (4°C). The total numbers of parasites within 15 fields of view (Objective 40X) were counted and compared between *mic2* KO and WT. Mean values of three independent experiments +/- SD were determined.

**Attachment under fluidic shear stress: **Fresh extracellular parasites (4 × 10 ^5^ in total consisting of approximately equal numbers of control and KO) were loaded into collagen IV coated fluidic chambers (Ibidi IB-80192) and allowed to attach at 37°C for 20 minutes. PBS was pumped through the chamber using an “open loop flow” microfluidic pump (KD Scientific Legato 200 syringe pump) system, similar to that described by ( Harker *et al.*, 2014), to control flow rates and generate fluidic shear stress. In our setup, a flow rate of 1 ml/min achieves 3 dyn/cm ^2^ shear stress at the surface of the channel. Flow at 0.1 ml/min (equivalent to 0.3 dyn/cm ^2^) was used to remove all non-attached parasites. At each fluidic shear stress level, control and mutant parasites were counted from 5 fields of view per experiment. Parasite count after the 0.1 ml/min wash was taken as 100% of attached parasites. Counts at all other rates of flow were normalised to the 100%. Data collected was analysed using Excel to assess significance of differences between control and mutants using Student’s t-test and further analysed using GraphPad Prism v. 6.01 software to display data as trends. Parasites in the chamber were monitored via a Zeiss Axio Vert.A1 microscope setup with a 40x objective combined with an AxioCam ICm1 camera and Zen capture software. Mean values of three independent experiments +/- SD were determined.

**Trail deposition assay:** Gliding assays were performed as described previously ( Håkansson *et al.*, 1999). Briefly, freshly lysed parasites were allowed to glide on FBS-coated glass slides for 30 min before they were fixed with 4% PFA and stained with α-SAG1 under non-permeabilising conditions. Mean values of three independent experiments +/- SD were determined.

**2D video motility assay:** Time-lapse video microscopy was used to analyse the kinetics over a 2D surface similar as previously described. Briefly a glass-bottom live cell dish (Ibidi μ-dish ^35mm-high^) was coated in 100% FBS for 2 hours at room temperature. Freshly egressed parasites were added to the dish. Time-lapse videos were taken with a 20X objective at 1 frame per second using a DeltaVision ^®^ Core microscope. Analysis was done using Fiji version 1.8.0_66 with the wrMTrckr version 1.04 tracking plugin ( http://www.phage.dk/plugins/wrmtrck.html). For analysis, 20 parasites were tracked during both helical and circular gliding with the corresponding distance travelled, average and maximum speeds determined. Mean values of three independent experiments +/- SD were determined.

**3D motility assay: ** Tachyzoites were prepared and assayed as previously described ( Leung *et al.*, 2014). Three independent biological replicates, each with three technical replicates, were performed. Parameters calculated from 3D motility assays were analyzed using two-way ANOVA with Sidak’s multiple comparisons test, with GraphPad Prism v. 6.01. Where statistically significant, multiplicity adjusted P values for comparisons are indicated with asterisks.

**Invasion and replication assay: ** 5×10 ^4^ freshly lysed parasites were allowed to invade a confluent layer of HFFs for 1 hour. Subsequently, five washing steps were performed for removal of extracellular parasites. Cells were then incubated for a further 24 hours before fixation with 4% PFA. Subsequently, parasites were stained with α-IMC1 antibody.

For invasion, the number of vacuoles in 15 fields of view (Objective 40X) was counted. Invasion rates were normalised to RH *Δhxgprt* at 100%. For replication, 200 vacuoles were counted for the number of parasites per vacuole. Mean values of three independent experiments +/- SD were determined.

**Red/Green assay: ** Classical “red/green” assays were performed as previously described by Huynh & Carruthers, 2006 to determine the percentage of invasion, independent of the attachment defect of *mic2* KO. 1 ×10 ^6^ parasites were allowed to invade a confluent monolayer of HFF cells for 1 hour. Extracellular parasites were stained with α-SAG1 under non-permeabilising conditions. For both strains ( *mic2* KO and *WT*) independently of the other, the number of invaded parasites was compared to the total number of parasites observed (attached + invaded), allowing us to mitigate the attachment phenotype of *mic2* KO. Mean values of three independent experiments +/- SD were determined.

**Junction formation:** 1× 10 ^6^ parasites were artificially released from their vacuole and allowed to invade for 10 minutes, after which the media was removed and 4% PFA was added, fixing the parasites mid-penetration. Coverslips were blocked under non-permeabilising conditions and stained for the rhoptry neck protein, RON2, and AMA1.

**Penetration time of invading parasites:** Freshly egressed parasites were added to a confluent monolayer of HFFs grown on a glass-bottom live cell dish (Ibidi μ-dish ^35mm-high^). Time-lapse images were taken at 1 image per second using a 40X objective in DIC for both RH *Δhxgprt* and *mic2* KO parasites. For penetration times, 20 invasion events were analysed and scored from the initial start point of a visible junction to complete parasite internalisation.

**Egress assay:** Egress assays were performed as previously described ( Black *et al.*, 2000). Briefly, 5×10 ^4^ parasites were grown on HFF monolayers for 36 hours. Media was exchanged for pre–warmed, serum–free DMEM supplemented with 2 µM A23187 (in DMSO) to artificially induce egress. After 5 minutes the cells were fixed with 4% PFA and stained with anti-SAG1 antibody without detergent permeabilization. 200 vacuoles were counted for parasite egress. Mean values of three independent assays +/- SEM were determined.

### *In vivo* infection model

All animal procedures conformed to guidelines from The Home Office of the UK Government under the Animals [Scientific Procedures] Act 1986. All work was covered by Licence PPL60/3929, “Mechanism of control of parasite infection” with approval by the University of Strathclyde ethical review board. BALB/c mice were bred in house at the Strathclyde Institute of Pharmacy and Biomedical Sciences, Glasgow, UK under specific pathogen free conditions. Mice were housed in polypropylene cages (13cm×35cm), containing Ecopure flakes and sizzle nest bedding (SDS Services) with access to water and CRM mouse chow (SDS Services) *ad libatim.* Care was taken to minimise suffering through provision of water soaked mouse chow. The minimum number of mice were used to give reliable qualitative results. Six to eight week old female mice (13.4–17.6g, mean 16.1g), grouped in cages of five, were used for infection studies. Mice were assigned randomly to groups by an independent worker with no knowledge of their experimental purpose. Prior to infection, all mice were weighed and subsequently monitored daily for morbidity and weight loss. Mice were euthanised when they reached the humane endpoints set out in the licence.

In initial phenotype studies, groups of five mice were each infected with 2×10 ^4^ WT or *lox*P *mic2* control or *mic2* KO tachyzoites in 200μl sterile PBS via intraperitoneal injection (IP). *In vivo* parasite burden was followed by bioluminescent imaging using *lox*P *mic2* and mic2 KO expressing firefly luciferase. Mice were infected with 2×10 ^4^ tachyzoites via intraperitoneal injection in a volume of 400μl sterile PBS between 10.00–12.00 hrs). For imaging, the mice were dosed with 150mg/kg D-luciferin potassium salt solution (PerkinElmer), anesthetised with isoflurane and imaged (between 10.00 and 12.0 hrs) using an IVIS Spectrum (PerkinElmer). Isoflurane was used as this is the standard and recommended procedure by PerkinElmer the manufacturer of the IVIS. One minute exposures were taken twenty minutes post luciferin injection. Radiance data were quantified using Living Image software 4.0 (Perkin Elmer) and statistical significance determined by Mann-Whitney test.

## Data availability

The data referenced by this article are under copyright with the following copyright statement: Copyright: © 2017 Gras S et al.

Complete Western blots for Figure 1 and Figure S1; raw count for all the assays (Plaque size, Attachment, Invasion, Replication, Egress, Flow, 2D video); raw galaxy results for *ama1* KO-RH comparison; raw galaxy results for *mic2* KO-RH comparison; raw galaxy results for *lox*P *mic2*- *mic2* KO comparison are available on OSF: DOI, 10.17605/OSF.IO/FASQG ( Meissner, 2017; https://osf.io/fasqg/).

## Supplementary material

**Figure S1: Loss of *mic2* impacts localization and secretion of M2AP, but not other micronemal proteins.** **A**) Localization by IFA of MIC proteins in *mic2* KO and WT tachyzoites; only M2AP localisation is altered. Scale bar: 2 μm. n=3, total number of vacuoles observed >300 **B**) *mic2* KO and WT tachyzoites were incubated in the absence (- ; constitutive secretion) or presence (+ ; induced secretion) of calcium ionophore, and the amount of MIC4 and M2AP secreted into the culture supernatant was determined by western blotting. Actin was used as a loading control. Black arrowhead indicates proM2AP and the empty arrowheads the processed forms ( Huynh & Carruthers, 2006); note that proM2AP is not properly processed in the *mic2* KO.

**Figure S2: Mean FPKM of MICs in the different KO strains.** Graphic representation of the mean FPKM value variation between RH (White), *lox*P *mic2* (Black), *mic2*KO (Red) and *ama1*KO (Yellow) strains. The difference between each mutant and RH was calculated using CutDiff with a comparison of three independent biological replicates, using quartile library normalization method, a dispersion estimation method using “pooled” with the tree replicate and a false discovery rate of 0.05. Statistically significant differences are indicated by *. Error bar indicates the FPKM standard deviation within the replicate. MIC5* FPKM value was divided by 2 for graphical purpose.

**Figure S3: Kinetic analysis of 2D circular gliding.** Kinetic analysis obtained from live records of parasites undergoing circular gliding. Data were analyzed using Analysis was done using Fiji with the wrMTrckr version 1.04 tracking plugin ( http://www.phage.dk/plugins/wrmtrck.html). Mean values of three independent assays are shown ± SEM. ***: p-value <0.001 in a two-tailed Student's t-test.

**Supplementary Videos: Representative gliding, invading and egressing parasites.** Video S1–S6, *mic2* KO and RH parasites making circular, helical and twirling motion; Video S7–S8, *mic2* KO and RH parasites invading host cells; Video S9–S10 *mic2* KO and RH parasites egressing from host cells. Video were recorded at 1 frame per second.

**Supplementary Tables: RNA seq data and primer list.** Table S1, RNA sequencing FPKM value of MICs in all strains (RH, *lox*P *mic2*, *mic2* KO, *ama1* KO). Table S2, RNA sequencing Accepted Hits between *mic2* KO and RH. Table S3, RNA sequencing Accepted Hits between *ama1* KO and RH. Table S4, Primers list used in the study. Table S5, antibodies used in the study. RH FPKM, MIC2 KO FPKM and AMA1 FPKM, contain the FPKM of the three triplicates for all Toxoplasma genes for RH, *mic2* KO and *ama1* KO strains. MIC2KO-RH and AMA1KO-RH total results obtained after Cutdiff analysis between RH and mutants.
