# Supplementary figures and images for: Parasites lacking the micronemal protein MIC2 are deficient in surface attachment and host cell egress, but remain virulent in vivo
Source: Wellcome Open Res. 2017 Jul 24;2:32. Originally published 2017 May 19. [Version 2] doi: 10.12688/wellcomeopenres.11594.2 (PMC5473411; doi:10.12688/wellcomeopenres.11594.2)

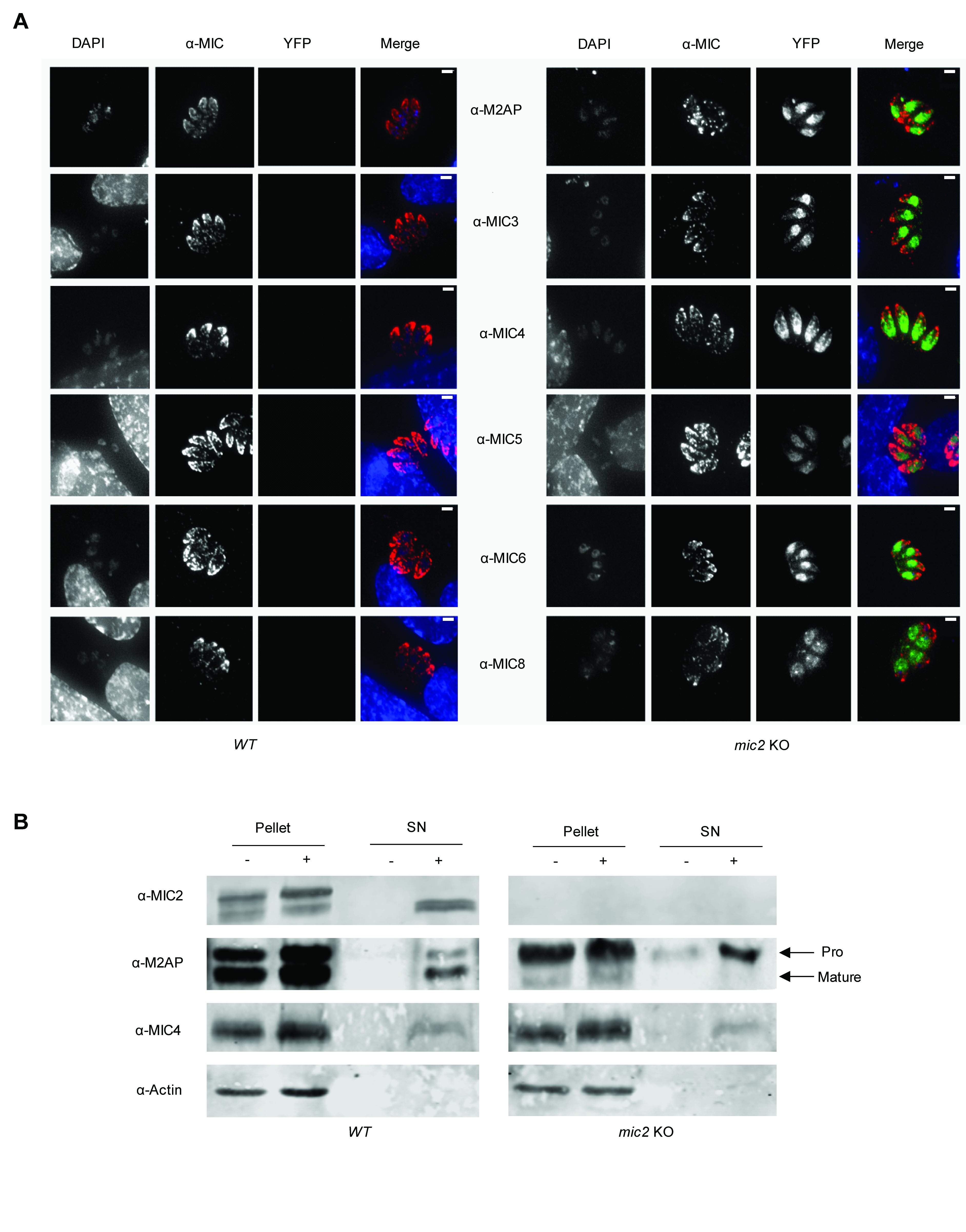

Supplement: Supplementary file 1 [file wellcomeopenres-2-13129-s0000.tgz › f25b59ca-66ac-4cc0-9fd1-2ffa3513ca3f.tif]

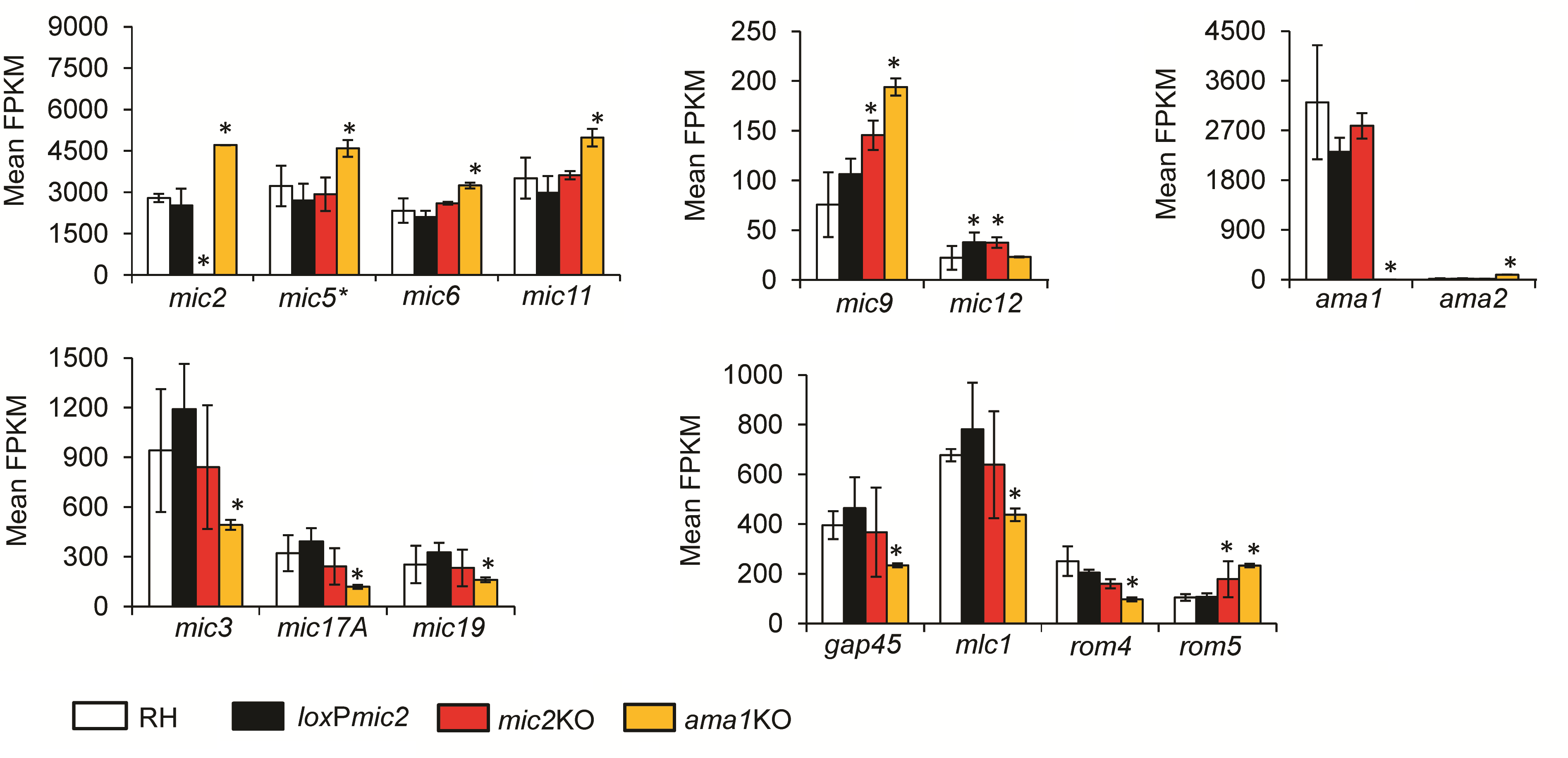

Supplement: Supplementary file 2 [file wellcomeopenres-2-13129-s0001.tgz › b6eaf310-2b7a-4e37-9627-300cc0c23ebe.tif]

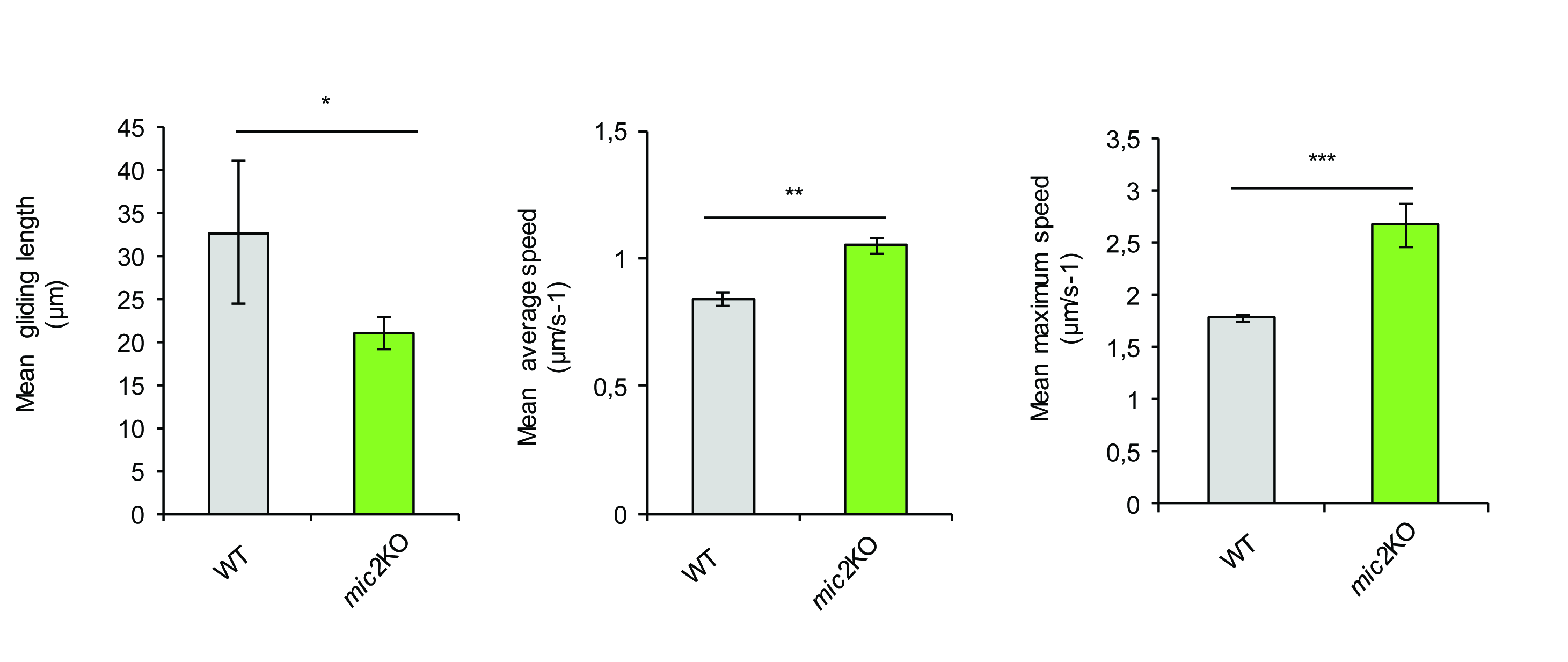

Supplement: Supplementary file 3 [file wellcomeopenres-2-13129-s0004.tgz › 43fc0416-5648-4be8-bf4e-eeb28c8a78dd.tif]
