# Supplementary material for: Parasites lacking the micronemal protein MIC2 are deficient in surface attachment and host cell egress, but remain virulent in vivo
Source: Wellcome Open Res. 2017 Jul 24;2:32. Originally published 2017 May 19. [Version 2] doi: 10.12688/wellcomeopenres.11594.2 (PMC5473411; doi:10.12688/wellcomeopenres.11594.2)
Supplement: Peer review history

# Peer review: Referee response for version 2

**DOI:** 10.21956/wellcomeopenres.13129.r24448

**Published:** 2017-08-02

**Authors:** Sebastian Lourido

**Affiliations:** 1 Whitehead Institute for Biomedical Research, Cambridge, MA, USA

The authors have addressed all of the concerns I previously raised. In particular, they now provide the complete reports for their RNAseq experiments, which will allow other investigators to recreate the analyses for themselves. I have no further comments on the manuscript.

I have read this submission. I believe that I have an appropriate level of expertise to confirm that it is of an acceptable scientific standard.

# Peer review: Referee response for version 2

**DOI:** 10.21956/wellcomeopenres.13129.r24449

**Published:** 2017-08-02

**Authors:** Vernon Carruthers, My-Hang Huynh

**Affiliations:** 1 Department of Microbiology and Immunology, University of Michigan School of Medicine, Ann Arbor, MI, 48109, USA; 2 Department of Microbiology and Immunology, University of Michigan School of Medicine, Ann Arbor, MI, USA

The authors have responded thoroughly to comments on the original submission and have revised the article in exemplary fashion. The article is now recommended for approval without further changes.

We have read this submission. We believe that we have an appropriate level of expertise to confirm that it is of an acceptable scientific standard.

# Peer review: Referee response for version 1

**DOI:** 10.21956/wellcomeopenres.12524.r22938

**Published:** 2017-06-12

**Authors:** Tim W. Gilberger, Paul-Chrisian Burda

**Affiliations:** 1 Bernhard Nocht Institute for Tropical Medicine, Hamburg, Germany; 2 Centre for Structural Systems Biology, Hamburg, Germany; 3 University of Hamburg, Hamburg, Germany

This well performed study by Gras and co-workers investigates the role of MIC2 in the apicomplexan parasite *Toxoplasma gondii*. The authors used a DiCre based knock-out cell line to analyze i) transcriptional responses, ii) host cell attachment, iii) gliding, iv) egress and v) virulence in mice.

 In summary, this manuscript provides a substantial body of novel insight into MIC2 function in *T. gondii* using state-of-the art approaches. It leaves us only with a few comments:

- Figure 3B: Please show SD also for the 0.5 dyn/cm ^2^ values
- Figure 4A+C, Figure 6D: Please include scale bar information in the figure legend.
- Fig. 4: Did the authors used a Students T-test (as described in the figure legend) or a two-way-Anova with Sidak’s multiple comparisons test as indicated in the Materials & Methods?
- Figure 5C: This figure is confusing and not well embedded in the result section. It would be helpful to include co-localization of AMA1 and RON2 in the *mic*2 knock out cell line. Additionally, they could provide the information of the YFP expression in WT *vs* KO.
- Figure 5D: The "ns" on top of the graphs is hardly readable
- Figure 7A: The black line for "Killed RH" is not visible
- Figure S3: The Y-Axis description is missing for the second and third data set
- The supplementary movie file cannot be opened

 

 Additional remarks:

- Abstract – “MIC2 appears to not be critical for gliding motility …”. This is correct, but in the light of Fig. 4A, showing a 60% reduction in gliding motility, a re-phrasing of this sentence might be good to avoid confusion.
- Page 5: The up-regulation of various invasion-related genes in both the *mic*2 KO and the AMA1 KO could be -as suggested- connected to multifactorial compensation, although this has to analyze in detail and compared to additional KO strains lacking other invasion-related proteins. An alternative explanation could be that the “general” up-regulation is “noise” due to the perturbation of the functional protein network.

We have read this submission. We believe that we have an appropriate level of expertise to confirm that it is of an acceptable scientific standard.

# Response

**Published:** 2017-07-10

**Authors:** Markus Meissner

**Affiliations:** University of Glasgow, UK

We thank the reviewers for their valuable comments, which helped us to improve our manuscript. We addressed all comments in our revision. See our response to the reviewers comments below (in bold) 

Figure 3B: Please show SD also for the 0.5 dyn/cm ^2^ values

**The value at 0.5 dyn/cm2 value have been set for each experiment to 100% to be our starting point and allow us to visualise the variation under stronger flow. By doing so, no SD can be calculated for this point**

Figure 4A+C, Figure 6D: Please include scale bar information in the figure legend.

**Information now included for all 3 panels**

Fig. 4: Did the authors used a Students T-test (as described in the figure legend) or a two-way-Anova with Sidak’s multiple comparisons test as indicated in the Materials & Methods?

**A two-way-Anova with Sidak’s multiple comparisons test as indicated in the Materials & Methods was used. The is now corrected in the text.**

Figure 5C: This figure is confusing and not well embedded in the result section. It would be helpful to include co-localization of AMA1 and RON2 in the *mic*2 knock out cell line. Additionally, they could provide the information of the YFP expression in WT *vs* KO.

**RON2 was used as a marker for the tight junction and was used ofr both *WT *and *mic2 KO *parasites to demonstrate normal junction formation. Ama-1 is only shown for WT to outline the parasite. In case of *mic2KO *parasites YFP expression is activation upon induction of DiCre, requiring no additional stain**.

Figure 5D: The "ns" on top of the graphs is hardly readable

**We changed this in the revision.**

Figure S3: The Y-Axis description is missing for the second and third data set

**We changed this in the revision.**

The supplementary movie file cannot be opened

**We have checked the files and they could be opened on our system.**

Additional remarks:

Abstract – “MIC2 appears to not be critical for gliding motility …”. This is correct, but in the light of Fig. 4A, showing a 60% reduction in gliding motility, a re-phrasing of this sentence might be good to avoid confusion.

**The sentence now reads “We verified the role of MIC2 in efficient surface attachment, but were unable to identify any direct function of MIC2 in sustaining gliding motility or host cell invasion once initiated.”**

Page 5: The up-regulation of various invasion-related genes in both the *mic*2 KO and the AMA1 KO could be -as suggested- connected to multifactorial compensation, although this has to analyze in detail and compared to additional KO strains lacking other invasion-related proteins. An alternative explanation could be that the “general” up-regulation is “noise” due to the perturbation of the functional protein network.

**We agree with the reviews that a more thorough analysis of transcriptional changes in different null mutants for components of the invasion machinery is worthwhile for future investigations.**

# Peer review: Referee response for version 1

**DOI:** 10.21956/wellcomeopenres.12524.r22939

**Published:** 2017-06-12

**Authors:** Sebastian Lourido

**Affiliations:** 1 Whitehead Institute for Biomedical Research, Cambridge, MA, USA

The present study by Gras *et al.* uses sophisticated phenotypic assays to dissect the role of MIC2 in the *Toxoplasma gondii* life cycle. Although the experiments are well executed and the conclusions are largely sound, the authors mainly recapitulate the discoveries of previous studies (Huynh & Carruthers. 2006; Harker *et al.* 2014). The current study differs in its use of a previously published direct knockout of MIC2 (Andenmatten et al. 2013), which clearly dispels many of the caveats of conditional knockout systems and demonstrates that MIC2 is not strictly essential for *T. gondii*. It is worth noting that the 2006 study concluded that MIC2 was a major determinant of invasion and virulence, but did not conclude that the gene was essential, consistent with the results presented by Gras *et al.*

 The new study identifies defects in attachment, gliding motility and invasion. It extends some of these observations further, by noting that the speed of motility and invasion are not altered, despite the shorter and less frequent tracks of movement and lower rates of invasion. Even under the simplistic models of gliding motility, a reduction in adhesins could have been predicted to reduce parasite attachment, which consequently would have reduced the strength, stability and persistence of movement. However, this study provides carefully crafted experiments to demonstrate these principles.

 By dividing egress between vacuole permeabilization and disruption the authors identify a delay in egress that may have been missed in the published analysis of the conditional knockout. Similarly, the authors clearly document lower levels of growth without MIC2 in the animal model, although mortality remains unchanged. At face value, these results differ from the previous report. However it was known that the background used for the conditional knockout what attenuated, and what supported the involvement of MIC2 in virulence in the Huynh and Carruthers study, was the sensitivity of the conditional knockout but not the parental strain to ATC (the agent used to downregulated MIC2 expression). The current study also showed diminished replication of MIC2 knockout in mice, however in their case this defect did not alter the ultimate virulence of the strain. While these discrepancies may reflect differences between the two studies, no mechanistic explanation is provided to account for the discordant results. In the absence of direct comparisons between the complete and conditional knockouts, such differences might represent variability in how the experiments are performed or in the basal virulence of the parasites used (as noted by the authors).

 Taken together, the current study provides a robust confirmation for the functions of MIC2 using the latest and most rigorous methods available in the field.

 Major concerns:

1. RNA sequencing results are not presented in their entirety. Such an analysis should contain a full account of the sequencing reads mapped so that readers can draw their own conclusions from the data. The authors should include a figure comparing the expression of all genes in the three strains (each mutant to WT, for example) and a supplementary file with the RPKM for each gene in each sample.
2. Page 5. The statement that “no single candidate [gene was] capable of compensating for the loss of MIC2” is not supported by the experimental data, since all the possible candidates have not been directly tested.
3. Page 10. The statement that “MIC2 might be involved in a signaling cascade that triggers host cell egress or parasite/parasite interaction” is not supported by the data, and should be omitted from the discussion. There is no evidence for a role of MIC2 in signaling and the lack of egress after vacuole rupture is most consistent with the 3D motility data that they present.

 

 Minor concerns:

1. Page 3. “a lower percentage of parasites devoid of MIC2 move for shorter distances” should read “a higher percentage of parasites” although the double negative is confusing.

I have read this submission. I believe that I have an appropriate level of expertise to confirm that it is of an acceptable scientific standard, however I have significant reservations, as outlined above.

# Response

**Published:** 2017-07-10

**Authors:** Markus Meissner

**Affiliations:** University of Glasgow, UK

We thank the reviewer for the comments, which helped to improve the manuscript. Please see our response below (in bold).

RNA sequencing results are not presented in their entirety. Such an analysis should contain a full account of the sequencing reads mapped so that readers can draw their own conclusions from the data. The authors should include a figure comparing the expression of all genes in the three strains (each mutant to WT, for example) and a supplementary file with the RPKM for each gene in each sample.”

**We initially attempted to make an overview figure, as suggested by the reviewer in our initial submission. However, we found it rather distracting, since we were unable to observe any clear tendencies when comparing the transcriptomes of the different parasite strains. Instead, we decided to focus on known invasion related genes. We believe that adding a figure comparing the expression of all genes in the three strains (each mutant to WT, for example) would not help in answering the question if redundancies/adaptation exist in response to deletion of *mic2*. To allow interested readers to access all data, we added RPKM of all genes cutdiff between WT/mic2 and WT/AMA1 to the supplementary information.**

Page 5. The statement that “no single candidate [gene was] capable of compensating for the loss of MIC2” is not supported by the experimental data, since all the possible candidates have not been directly tested.

**The sentence now reads: "However, based on this analysis we were unable to identify a clear candidate that could compensate for los of *mic2.*"**

Page 10. The statement that “MIC2 might be involved in a signaling cascade that triggers host cell egress or parasite/parasite interaction” is not supported by the data, and should be omitted from the discussion. There is no evidence for a role of MIC2 in signaling and the lack of egress after vacuole rupture is most consistent with the 3D motility data that they present.

**We agree with the reviewer that at this point it is a speculation and we decided to remove this statement in the discussion.**

Minor concerns:

“Page 3. “a lower percentage of parasites devoid of MIC2 move for shorter distances” should read “a higher percentage of parasites” although the double negative is confusing.”

**The sentence now reads “However, while *mic2*KO parasites exhibited less motility and move on shorter distance, they reach the same maximal speeds as WT parasites” for more clarity.**

# Peer review: Referee response for version 1

**DOI:** 10.21956/wellcomeopenres.12524.r22936

**Published:** 2017-06-09

**Authors:** Vernon Carruthers, My-Hang Huynh

**Affiliations:** 1 Department of Microbiology and Immunology, University of Michigan School of Medicine, Ann Arbor, MI, 48109, USA; 2 Department of Microbiology and Immunology, University of Michigan School of Medicine, Ann Arbor, MI, USA

The Toxoplasma microneme protein MIC2 has been a central figure in the widely, albeit not uniformly, accepted model of gliding motility and active cell invasion. Previous work using a Tet-off conditional knockdown mutant suggested that MIC2 contributes substantially to gliding motility and attachment, which together result in an invasion defect ^1^. The current study largely corroborates the earlier findings, and includes additional analyses that more thoroughly establish a role for MIC2 in initiating gliding motility in both 2D and 3D gliding assays. Importantly, the study also includes assessment of transcriptional changes in MIC2 knockout parasites. Overall, data in the current study is of high quality, well controlled, and the phenotypic assessments are thorough and robust for most experiments. The conclusions are also generally well supported by the data, and the article is well written and accessible to a wide audience. Addressing the following aspects will further improve the study.

  

1. The most striking apparent difference between the conditional knockdown and the knockout is seen in infected mice. Infection studies with the conditional knockdown required injection of a relatively high inoculum of 50,000 tachyzoites to overcome the intrinsic virulence deficiency of the Tet regulated (Tati) strain, a fact that is discussed in the current study as the potential basis for the apparent discrepancy in assessment of virulence. 50,000 Tati parasites is the minimum dose needed to consistently observe lethal infection. Attaining lethal infection in 33% of mice infected with MIC2 conditional knockdown parasites required injection of 3x107 tachyzoites, which is 625 times higher than for the Tati strain. The current study uses a non-Tati strain that is presumably equally virulent to the RH strain, although this was not evaluated. RH strain has an effective LD100 of 1 viable tachyzoite via IP injection. However, assessment of virulence in this study was conducted by injecting 1,000 tachyzoites, which is 1000x the LD100. Most studies assess the virulence of mutants in an RH strain background by injecting 10-100 tachyzoites, and several more recent studies have utilized subcutaneous injection because this route renders it easier to detect a virulence deficiency. Accordingly, the conclusion that MIC2 knockout parasites have a minor virulence defect is not substantiated by the data as it currently stands without a more thorough assessment with lower inocula. In the absence of such an assessment, because a 1000x LD100 dose was used in the current study it theoretically remains possible that MIC2 knockout parasites have a loss of virulence that is similar or equal to that of MIC2 knockdown parasites. The authors should either address this aspect with additional experiments, or acknowledge this possibility in the discussion. Also, it appears that the virulence experiment was only performed once, and with only 5 mice per group. Thus, reproducibility was not established, and the experiment is underpowered.
2. The authors conclude that MIC2 knockout parasites have a similar distribution in infected mice as WT parasites. There are two limitations to this conclusion. First, the resolution of whole body bioluminescence is very poor, and thus does not allow one to distinguish between organs unless the organs are removed and imaged individually. Second, IP infection with RH strain parasites results mostly in intraperitoneal replication, with limited dissemination prior to mortality. The authors should assess bioluminescence in individual organs or acknowledge  limitations of using this approach and the strain in assessing dissemination.
3. The caption for Figure 4 states: “Maximal speed of gliding is not impacted by mic2 KO”. This is somewhat misleading because it describes the findings of only 20% of the data in the figure. The figure has 10 panels or graphs, 8 of which show differences in gliding between WT and mic2 KO parasites. The figure caption should describe both the differences and the similarities.
4. Please provide y-axis labels for the middle and right panels of Fig S3. Also, for the sentence: “Surprisingly, the average and maximum speeds increased compared to WT parasites (Figure S3)” it should be explicitly indicated that this is for circular gliding.
5. Please describe in the materials and methods how the virulence experiment shown in Fig 7A was performed, including stating the number of times the experiment was performed and the number of animals in the experiment. Also, indicate in the legend of Fig 7 the inoculum for the bioluminescence experiment, and state in the methods the number of times the experiment was performed along with the number of mice used.
6. Examples shown in Fig 6D do not match the categories used for quantification in Fig. 6C. The authors should consider showing an example of an intact PV instead of the “Lysed” example. Also, it would be ideal to explicitly state in the materials and methods that for assessing egress the fixed monolayers were not detergent permeabilized.

We have read this submission. We believe that we have an appropriate level of expertise to confirm that it is of an acceptable scientific standard, however we have significant reservations, as outlined above.

## References

[1] HuynhMHCarruthersVB: Toxoplasma MIC2 is a major determinant of invasion and virulence. *PLoS Pathog*.2006;2(8) : 10.1371/journal.ppat.0020084 e84 10.1371/journal.ppat.0020084 16933991PMC1550269

# Response

**Published:** 2017-07-10

**Authors:** Markus Meissner

**Affiliations:** University of Glasgow, UK

**We would like to thank reviewer three for his comments. We share some of his concerns regarding virulence in mice and pointed this out more clearly in our revision. See our detailed response below (in bold):**

The most striking apparent difference between the conditional knockdown and the knockout is seen in infected mice. Infection studies with the conditional knockdown required injection of a relatively high inoculum of 50,000 tachyzoites to overcome the intrinsic virulence deficiency of the Tet regulated (Tati) strain, a fact that is discussed in the current study as the potential basis for the apparent discrepancy in assessment of virulence. 50,000 Tati parasites is the minimum dose needed to consistently observe lethal infection. Attaining lethal infection in 33% of mice infected with MIC2 conditional knockdown parasites required injection of 3x107 tachyzoites, which is 625 times higher than for the Tati strain. The current study uses a non-Tati strain that is presumably equally virulent to the RH strain, although this was not evaluated. RH strain has an effective LD100 of 1 viable tachyzoite via IP injection. However, assessment of virulence in this study was conducted by injecting 1,000 tachyzoites, which is 1000x the LD100. Most studies assess the virulence of mutants in an RH strain background by injecting 10-100 tachyzoites, and several more recent studies have utilized subcutaneous injection because this route renders it easier to detect a virulence deficiency. Accordingly, the conclusion that MIC2 knockout parasites have a minor virulence defect is not substantiated by the data as it currently stands without a more thorough assessment with lower inocula. In the absence of such an assessment, because a 1000x LD100 dose was used in the current study it theoretically remains possible that MIC2 knockout parasites have a loss of virulence that is similar or equal to that of MIC2 knockdown parasites. The authors should either address this aspect with additional experiments, or acknowledge this possibility in the discussion. Also, it appears that the virulence experiment was only performed once, and with only 5 mice per group. Thus, reproducibility was not established, and the experiment is underpowered.

The authors conclude that MIC2 knockout parasites have a similar distribution in infected mice as WT parasites. There are two limitations to this conclusion. First, the resolution of whole body bioluminescence is very poor, and thus does not allow one to distinguish between organs unless the organs are removed and imaged individually. Second, IP infection with RH strain parasites results mostly in intraperitoneal replication, with limited dissemination prior to mortality. The authors should assess bioluminescence in individual organs or acknowledge limitations of using this approach and the strain in assessing dissemination.

**We agree with the reviewer that the results need to be interpreted with care and changed our discussion according to the reviewers suggestions. It reads now:**

**“Most surprisingly, and in contrast to the results obtained for the MIC2 conditional knockdown ( Huynh & Carruthers, 2006), we find that mic2 KO parasites are only mildly attenuated compared to WT RH parasites. The mic2 KO line (at least at the doses used in these studies) induces lethal disease in BALB/c mice.The mic2 KO line induces lethal disease in BALB/c mice, and transfection of these parasites with a luciferase gene demonstrated that they grow in similar anatomical locations in mice as control parasites. One explanation for the different findings could be that RH TATi ΔHX, the parasite line used to generate a knockdown for mic2 is already severely attenuated due to the expression of the Tet-transactivator. Indeed, in the study by Huynh & Carruthers, 2006, high doses (5 × 10 4 tachyzoites) with RH TATi ΔHX were required to achieve normal time-to-death kinetics. It is thus possible that knockdown of mic2 in this strain reflects an enhancement of the already attenuated phenotype, while depletion in a WT background has only mild effects on parasite virulence. Alternatively, in our studies a reduced virulence of mic2KO parasites is not evident because of the relatively high numbers of tachyzoites used to facilitate in vivo imaging. Consequently to fully compare the virulence LD50 studies would be required. Nonetheless our studies demonstrate unequivocally that MIC2 is not necessary for in vivo infection and lethality in BALB/c mice. In vivo imaging and 3D diffuse tomographic reconstruction of mice infected with MIC2 deficient parasites transfected with a luciferase gene demonstrated that they grow in similar anatomical locations in mice as control parasites. However, dissection and histopathological analyses would be necessary to rule out any potential differences in tissue trophism."**

The caption for Figure 4 states: “Maximal speed of gliding is not impacted by mic2 KO”. This is somewhat misleading because it describes the findings of only 20% of the data in the figure. The figure has 10 panels or graphs, 8 of which show differences in gliding between WT and mic2 KO parasites. The figure caption should describe both the differences and the similarities.

**The capitation now read “Figure 4: Gliding initiation and gliding distance are affected by *mic2* KO attachment defect but not maximal speed”**

Please provide y-axis labels for the middle and right panels of Fig S3. Also, for the sentence: “Surprisingly, the average and maximum speeds increased compared to WT parasites (Figure S3)” it should be explicitly indicated that this is for circular gliding.

**Modification have been made and the text now reads “Surprisingly, the average and maximum speeds of circular gliding increased compared to WT parasites (Figure S3).”**

Please describe in the materials and methods how the virulence experiment shown in Fig 7A was performed, including stating the number of times the experiment was performed and the number of animals in the experiment. Also, indicate in the legend of Fig 7 the inoculum for the bioluminescence experiment, and state in the methods the number of times the experiment was performed along with the number of mice used.

**We updated the Material Methods section and describe the virulence experiments in greater detail.**

Examples shown in Fig 6D do not match the categories used for quantification in Fig. 6C. The authors should consider showing an example of an intact PV instead of the “Lysed” example. Also, it would be ideal to explicitly state in the materials and methods that for assessing egress the fixed monolayers were not detergent permeabilized.

**We changed Figure 6D as suggested and updated Material and Methods as suggested.**
